# Alzheimer’s disease-related transcriptional sex differences in myeloid cells

**DOI:** 10.1186/s12974-022-02604-w

**Published:** 2022-10-05

**Authors:** Isabelle Coales, Stergios Tsartsalis, Nurun Fancy, Maria Weinert, Daniel Clode, David Owen, Paul M. Matthews

**Affiliations:** 1 Department of Brain Sciences, Imperial College London, London, UK; 2 Department of Psychiatry, University of Geneva, Geneva, Switzerland; 3 UK Dementia Research Centre at Imperial College London, London, UK; 4 Present Address: Centre for Host Microbiome Interactions, King’s College London, London, SE1 9RT UK; 5 Hammersmith Hospital, E502, Burlington Danes Building, DuCane Road, London, W12 0NN UK; 6 Clinical Research Facility, Hammersmith Hospital, ICTM Building, DuCane Road, London, W12 0NN UK

**Keywords:** Microglia, Sex, Neurodegeneration, Neuroinflammation

## Abstract

Sex differences have been identified in many diseases associated with dysregulated immune responses, including Alzheimer’s disease (AD), for which approximately two-thirds of patients are women. An accumulating body of research indicates that microglia may play a causal role in the pathogenesis of this disease. We hypothesised that sex differences in the transcriptome of human myeloid cells may contribute to the sex difference observed in AD prevalence. To explore this, we assessed bulk and single-nuclear RNA sequencing data sets generated from four human derived myeloid cell populations: post-mortem microglial nuclei, peripheral monocytes, monocyte-derived macrophages (MDMs) and induced pluripotent stem cell derived microglial-like cells (MGLs). We found that expression of AD risk genes, gene signatures associated with the inflammatory response in AD, and genes related to proinflammatory immune responses were enriched in microglial nuclei isolated from aged female donors without ante-mortem neurological disease, relative to those from males. In addition, these inflammation-associated gene sets were found to be enriched in peripheral monocytes isolated from postmenopausal women and in MDMs obtained from premenopausal individuals relative to age-matched males. Expression of these gene sets did not differ in MDMs derived from women whose blood was sampled across the menstrual cycle or in MGLs cultured with 17β-oestradiol. This suggests that the observed gene set enrichments in myeloid cells from women were not being driven by acute hormonal influences. Together, these data support the hypothesis that the increased prevalence of AD in women may be partly explained by a myeloid cell phenotype biased towards expression of biological processes relevant to AD.

**Supplementary Information:**

The online version contains supplementary material available at 10.1186/s12974-022-02604-w.

## Introduction

Multiple lines of evidence suggest differences in both innate and adaptive immune responses between males and females [1, 2]. Relative to men, women clear some bacterial and viral infections more rapidly [1, 3–7], show a stronger immunogenic response to certain vaccinations [8], and have an increased risk of autoimmune diseases [2]. In addition, sex differences are found in many diseases associated with dysregulated immune responses [2]. This includes Alzheimer’s disease (AD), in which approximately twofold more women are being diagnosed with the disease compared to men—a difference which cannot be explained by disparities in life expectancy alone [9]. Accumulation of reactive microglia around amyloid plaques and tau aggregates are consistent neuropathological features of the disease [10]. Furthermore, genes associated with AD from genome-wide association studies (GWAS) are enriched in microglia, implicating them causally in disease susceptibility [11–16]. In the rodent brain, sex differences have been observed in microglial distribution [17, 18], morphology [17, 19], functional output [19–21], transcriptional profile [17, 18, 21–24], and immune responsiveness [22, 25–29]. For example, microglia from female mice exhibit augmented expression of an APOE-driven network of genes associated with aging, amyloidosis, and tau [30–32]. Yet, whilst peripheral human immune cells isolated from men and women show phenotypic and functional variations [1, 2], there are only limited studies examining sex differences in human microglia [33–35].

Sex-specific phenotypes may be partially explained by differences in exposure to sex hormones. For example, oestrogen exerts both pro- and anti-inflammatory effects on peripheral immune cells depending on the inflammatory stimulus, dose, and cell type [36]. Sexually dimorphic immune responses also could arise due to incomplete X-chromosome inactivation in females. Many genes contained on the X chromosome have been linked to immune-related functions [2, 37], and ~ 23% of X-linked genes retain augmented expression in females [38]. Given the central role of microglia in disease susceptibility and the sex-specific differences in microglial phenotype and function observed in mice, we hypothesised that the greater prevalence of AD in women may be partially explained by sex differences in microglia. To test this hypothesis, we sought to determine whether microglia isolated from women exhibit a transcriptional signature enriched for processes relevant to AD, in comparison with those from men.

## Results

### Alzheimer’s disease risk genes are enriched in post-mortem microglia from women

**Fig. 1:**
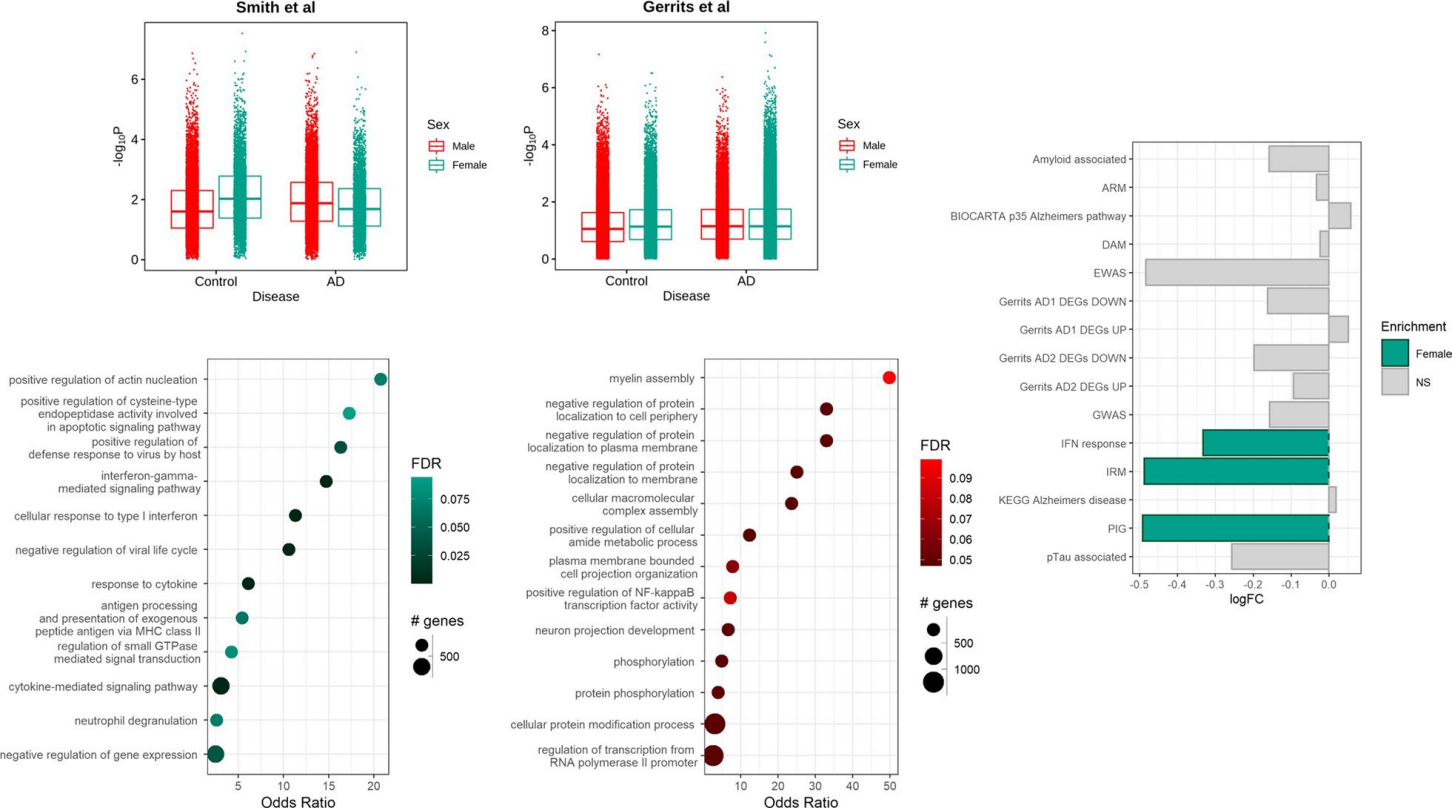
Sex differences in the transcriptome of post-mortem microglial nuclei isolated from non-disease controls. **A** Box plots showing the enrichment of the Alzheimer’s disease GWAS signal (− log10P) between the F-MGN and M-MGN from previously published data sets [39, 41]. **B**, **C** Dot plots showing the enriched gene ontology (GO) biological processes in the upregulated sDEG in the **B** F-MGN (blue) and **C** M-MGN (red) isolated post-mortem from non-diseased brains. Significance of sDEG was determined via MAST analysis using an FDR < 0.05. The size of the dots corresponds to the size of the gene set, whereas the intensity of the colour denotes the FDR value of the overrepresentation of the corresponding biological pathway in the sDEGs. **D** Bar chart showing the enrichment of IR-AD gene sets. A negative log fold change (logFC) shows enrichments in the female nuclei whilst a positive logFC denotes enrichment in males

To identify sex differences in microglial phenotypes, we re-analysed the transcriptome of microglia-enriched nuclei isolated from non-diseased control and AD brains previously generated in our lab (the discovery set, *n* = 27,592) [39]. These nuclei were isolated from two distinct brain regions to characterise transcript expression with both higher (entorhinal cortex) and lower (somatosensory cortex) tissue densities of neurofibrillary tangles and amyloid-beta plaques. First, we aimed to determine the presence of sex differences in the expression of genes associated with an increased risk of AD in microglia. Exploring the transcriptional enrichment of genes identified by GWAS as associated with a disease of interest provides a test for the heritability of the disease-relevant phenotype. In this case, in accordance with previous literature [40], we tested for the enrichment of sex-associated transcripts with AD GWAS genes to explore the differential heritability of AD-susceptibility related phenotypes in microglia between sexes. We observed a significant main effect of sex (*p* = 0.006) on the enrichment of the AD GWAS signal with post hoc analysis determining that this was accounted for by greater enrichment of AD GWAS loci-associated genes in microglial nuclei isolated from brains of women (F-MGN) relative to those from men (M-MGN). Moreover, this enrichment in F-MGN was greater in the non-diseased control brains relative (*p* = 0.008 for the Sex:Disease interation effect) to those with AD, where significance was not reached (*p* = 0.429) (Fig. 1A). We determined a similar significant enrichment of the AD GWAS signal in F-MGN relative to M-MGM (*p* = 0.030) in a larger, independent microglial-enriched snRNA-seq data set [41]. This difference was observed across nuclei isolated from both non-disease control and AD brains with no Sex:Disease interaction (*p* = 0.103).

### Inflammatory gene signatures associated with AD are enriched in microglia from post-menopausal women

Next, we tested for differentially expressed genes by sex (sDEG) in our control discovery microglia snRNA-seq data set using MAST [42]. A total of 409 sDEG were identified (Additional file 2: Table S1), of which 287 (70.2%) were upregulated in F-MGN and 122 (29.8%) in M-MGN. Enrichment analyses performed on genes significantly differentially expressed by F-MGN found they were associated with gene ontology (GO) biological processes relating to the immune response, including the response to type I interferon, antigen processing and presentation via MHC class I, and the response to cytokines (Fig. 1B). Genes differentially expressed in M-MGN were predominantly associated with pathways for the localisation and phosphorylation of proteins (Fig. 1C). Having established that many AD risk genes are overexpressed in F-MGN relative to M-MGN, we next tested whether F-MGN were enriched for inflammatory gene signatures previously associated with AD (Additional file 2: Table S2). This included both gene sets discovered using mouse models [29, 31, 43–46] and gene sets derived from studies of human patients. The latter included AD susceptibility genes identified via epigenome wide association studies (EWAS) [47, 48], alongside amyloid-β (AD1) and hyperphosphorylated-tau (AD2) associated phenotypes described in a snRNA-Seq analysis of *post-mortem* microglia [41].

We used gene set variation analysis (GSVA) [49] to calculate samplewise gene set enrichment scores in testing for enrichment of these inflammatory response in AD (IR-AD) gene sets in F- and M-MGN. F-MGN were enriched for IR-AD gene sets characterised by high expression of genes associated with interferon signalling pathways—the IRM (interferon responsive microglia) [31] and IFN-response [44] microglial phenotypes—as well as plaque-induced genes (PIG) [45]. None of the IR-AD gene sets tested were enriched in M-MGN (Fig. 1D). These sex-specific differences were found only in non-diseased donors; microglial nuclei from brains of donors with AD did not show significant differences between the sexes. Taken together, these data provide new evidence that microglia from non-diseased female donors are enriched for expression of inflammatory genes and pathways associated with AD relative to those from males.

### Monocytes from post-menopausal women exhibit enriched expression of gene sets for inflammatory responses related to AD

To test whether the enrichment of AD susceptibility genes and IR-AD gene sets was unique to F-MGN or whether a similar enrichment was also present in peripheral myeloid cells, we analysed a publicly available microarray data set (E-GEOD-56047) generated from human peripheral monocytes. To reduce the potentially confounding effect of the sex hormones, we only included samples from post-menopausal female donors (F-MCs) and aged matched males (M-MCs), as described in methods (*n* = 593) (Additional file 1: Fig. S1).

**Fig. 2:**
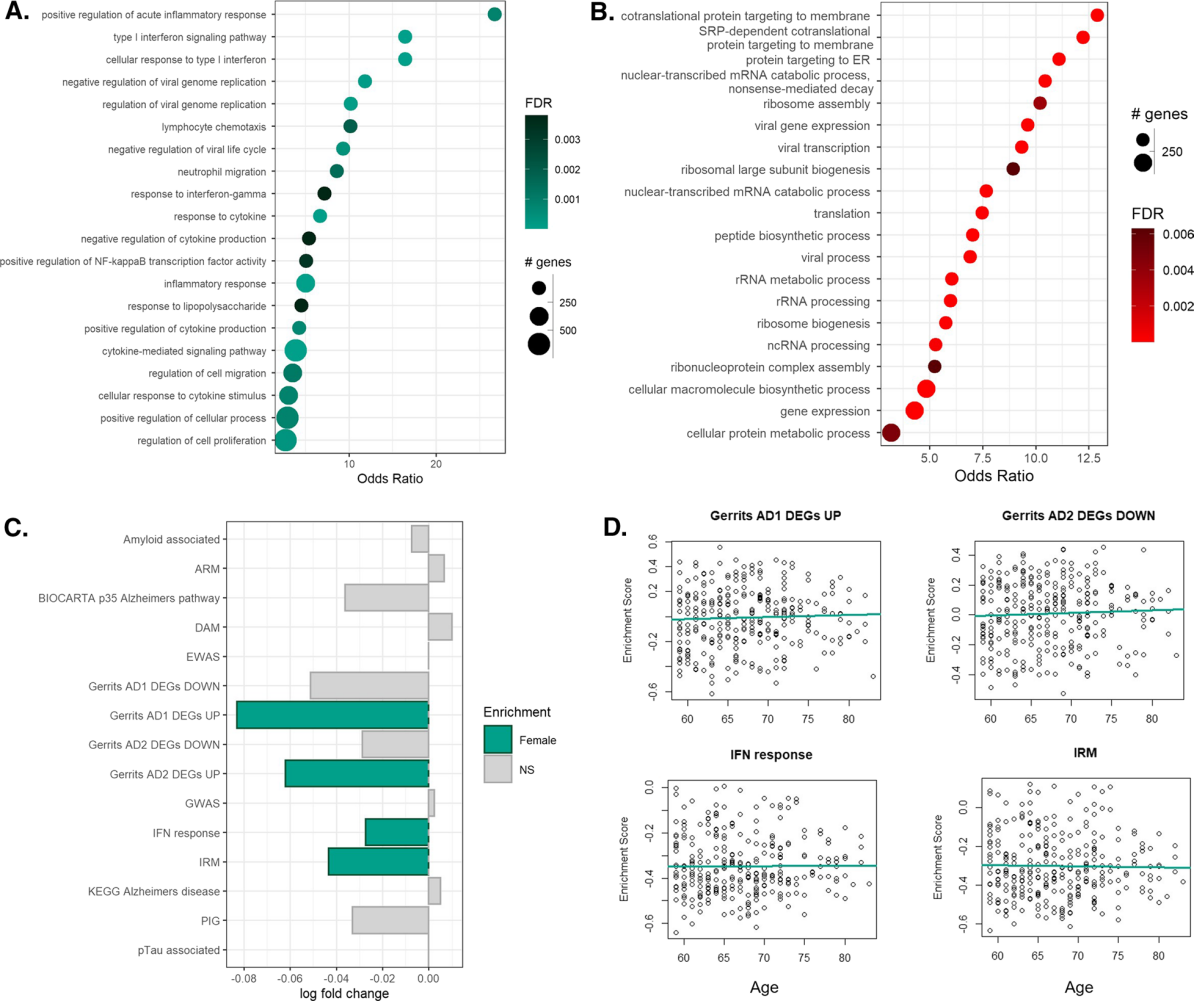
Sex differences in the transcriptome of aged ex vivo monocytes. **A**, **B** Dot plots showing the enriched gene ontology biological processes in the significantly sDEG upregulated in the nuclei of **A** F-MC (blue) and **B** M-MC (red). Significance of sDEG was determined via MAST analysis using an FDR < 0.05. **C** Bar chart showing the enrichment of IR-AD gene sets. A negative log fold change (logFC) denotes enrichment in F-MCs whilst a positive logFC denotes enrichment in M-MCs. **D** Correlation of the gene set enrichment scores for all IR-AD gene sets significantly enriched in the female samples with age. These data were derived from the re-analysis of the publicly available microarray data set E-GEOD-56047

From these data, we characterised genes differentially expressed in monocytes from women relative to those from men. We identified a total of 1301 sDEG, 690 and 611 of which were enriched in the F-MCs and M-MCs, respectively (Additional file 2: Table S3). Enrichment analyses of the genes upregulated in the F-MCs revealed sDEG overwhelmingly associated with GO terms relating to inflammation-associated pathways, including type I interferon signalling, positive regulation of NFκB activity, and response to lipopolysaccharide (Fig. 2A). In contrast, genes upregulated in M-MCs were associated with pathways for protein translation and trafficking (Fig. 2B).

We then tested the same set of monocytes data set for sex differences in enrichment for AD GWAS loci and IR-AD gene sets. Whilst significant sex differences in the enrichment of gene expression related to GWAS loci were not observed between monocytes from men and women, F-MCs were enriched for the expression of IRM [31] (*p* = 0.002) and IFN-response gene sets [44] (*p* = 0.006) and for both AD1 [41] (*p* < 0.001) and AD2 [41] (*p* = 0.002) upregulated-genes; none of the IR-AD gene sets were significantly enriched in the M-MCs (Fig. 2C). As AD is typically diagnosed around 75 years of age [9], we examined whether the expression of the IR-AD gene sets whose expression was enriched in monocytes from women were influenced by the age of the donor. No correlations with age were found for the enrichment of gene sets in the F-MC data (Fig. 2D); however, as the age range employed here was narrow (59–83 years) these results need to be confirmed in a larger population including a wider age range.

We, therefore, concluded that, like brain microglia from non-diseased women, monocytes from healthy postmenopausal women also are enriched for expression of inflammatory genes, including those for pathways associated with AD, relative to those isolated from healthy men of similar ages.

### Expression of gene sets for inflammatory responses to AD in myeloid cells from women is not modulated by exposure to female sex hormones

**Fig. 3:**
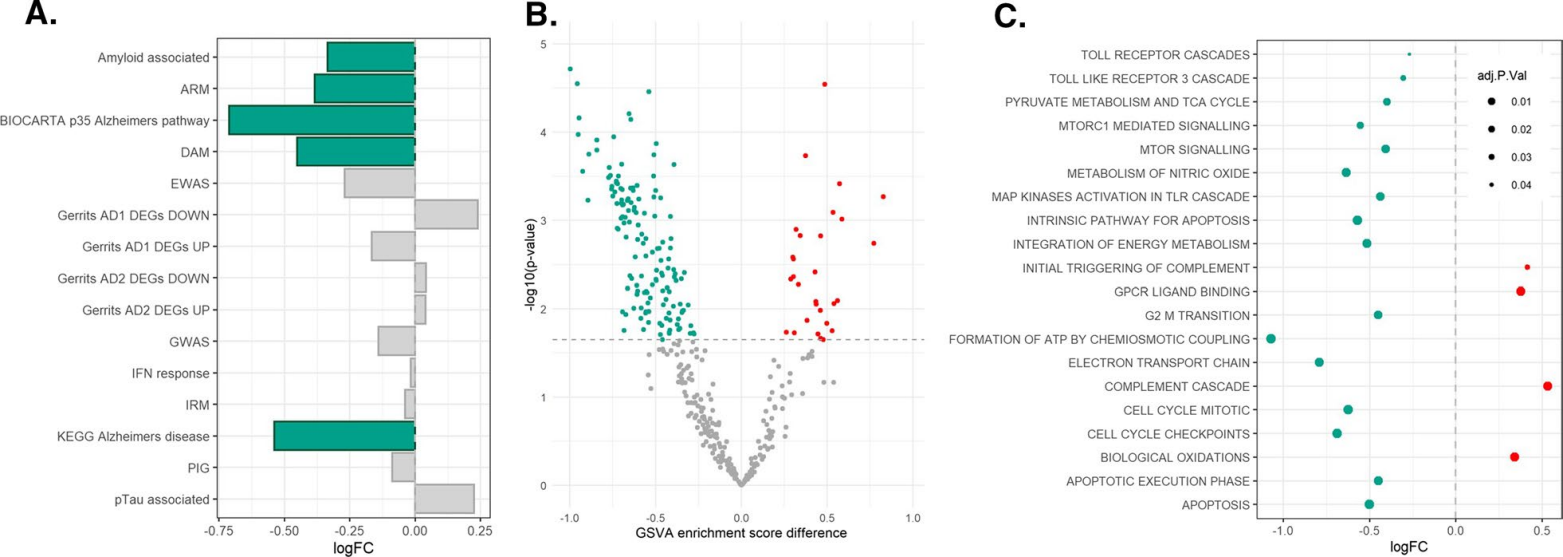
Sex differences in the transcriptomic profile of monocyte-derived macrophages from women (F_lo_) and men. **A** Bar chart showing the enrichment of IR-AD gene sets. A negative log fold change (logFC) denotes enrichment in F_lo_-MDMs whilst a positive logFC denotes enrichment in M-MDMs. Significantly enriched gene sets are shown in colour, with blue indicating enrichment in F_lo_-MDMs. **B** Volcano plot showing the number of significantly differently expressed Reactome and XiE (genes known to escape X-chromosome inactivation) gene sets between the M- and F_lo_-MDMs. Each dot represents one of the total 382 gene sets. **C** Dot plot showing a collection of significantly sexually differentially expressed gene sets (sDGS)  between M-MDMs and F_lo_-MDMs.  For plots **B** and **C**, p values were calculated via mixed-effects models with Benjamin-Hochberg multiple test corrections.

Having established that AD associated microglial gene sets are enriched in peripheral myeloid cells from post-menopausal women, we recruited pre-menopausal women to determine whether a similar enrichment was observed, and if so, whether the gene set enrichments were influenced by exposure to sex hormones. To explore these questions, peripheral monocytes were isolated from women at two points in their menstrual cycle. Firstly, during menstruation, when levels of circulating sex hormones are at their lowest (F_lo_), and then during the predicted late follicular phase of the cycle when levels of 17β-oestradiol (E2, the primary circulating oestrogen [50]) peak (F_hi_). Measurements of total serum E2 when blood was drawn for the monocyte isolations confirmed their associations with either F_lo_ or F_hi_ (Additional file 1: Fig. S2). Paired with those from age-matched men, the isolated monocytes were then differentiated in vitro into macrophages. None of the IR-AD gene sets were found to be significantly upregulated in the MDMs from men (M-MDMs) compared to those generated from monocytes of women sampled during menstruation (F_lo_-MDMs). However, the F_lo_-MDMs were relatively enriched for expression of amyloid-associated [43] (*p* = 0.020), ARM (activated response microglia) [31] (*p* = 0.019) and DAM (disease-associated microglia) [46] microglial phenotypes (*p* = 0.001), as well as a collection of genes associated with AD collated via the Biocarta (*p* = 0.026) and KEGG (*p* = 0.006) databases (Fig. 3A). To determine the influence of the menstrual cycle at the point of donation on the expression of IR-AD, we next compared their expression between F_lo_- and F_hi_-MDMs; however, no differences in the relative enrichment of these gene sets was observed (Additional file 2: Table S4).

In contrast to the analysis of MGNs and MCs, differential expression analyses comparing M- and F_lo_-MDMs identified only 26 sDEG (Additional file 2: Table S5). GSVA then was applied to explore sex differences in the enrichment of pathways collated via the Reactome database. This revealed 182 sexually differentially enriched gene sets (sDGS) between M- and F_lo_-MDMs (Fig. 3B; Additional file 2: Table S6). The sDGS enriched in F_lo_-MDMs (shown as a negative log fold change) included genes involved in immune response and programmed cell death (positive log fold change, Fig. 3C). sDGS enriched in M-MDM (shown as a positive log fold change) were associated with signalling by G-protein coupled receptors. No differentially expressed gene sets were identified via pairwise contrast of F_lo_- and F_hi_-MDMs (Additional file 2: Table S7) suggesting no effect of oestrogen exposure at the point of donation on the expression AD-related genes in MDM derived from pre-menopausal women.

We next sought to determine the direct effect of E2 on expression on IR-AD gene sets in iPSC derived microglia-like cells (MGLs) in vitro. For this, human iPSC lines were obtained from aged-matched healthy men and women, aged 60–64, and differentiated into MGLs [51]. Expression of genes encoding the nuclear oestrogen receptor α (ESR1) and the membrane bound oestrogen receptor GPER1, were confirmed in MGLs derived from both male and female donors (M-MGL and F-MGL, respectively) (Additional file 1: Fig. S3). Whilst the experiment was not powered to detect sex differences in the non-E2 groups, comparisons of genes expressed in naïve M- and F-MGLs found that, prior to multiple test corrections, F-MGLs were enriched for both IRM [31] (*p* = 0.016) and IFN-response [44] (*p* = 0.026) microglial phenotypes, consistent with our findings from both *post-mortem* microglia and peripheral monocytes. Subsequently, they were exposed to E2 for 24 h. There was no difference in the enrichment of IR-AD gene sets between the naïve and E2-treated MGLs in either sex, further suggesting little or no influence of exposure to E2 on their expression.

Together, these analyses confirmed that sex differences in the enrichment of IR-AD gene sets were found in MDM and that neither in vitro exposure to oestrogen, nor sampling at time of high and low E2 in the menstrual cycle, appears to modulate the relative enrichment of IR-AD gene sets in peripheral myeloid cells from women.

### Leading edge genes enriched in myeloid cells and microglia from women are related to those differentially expressed in microglia isolated from individuals with AD

**Fig. 4:**
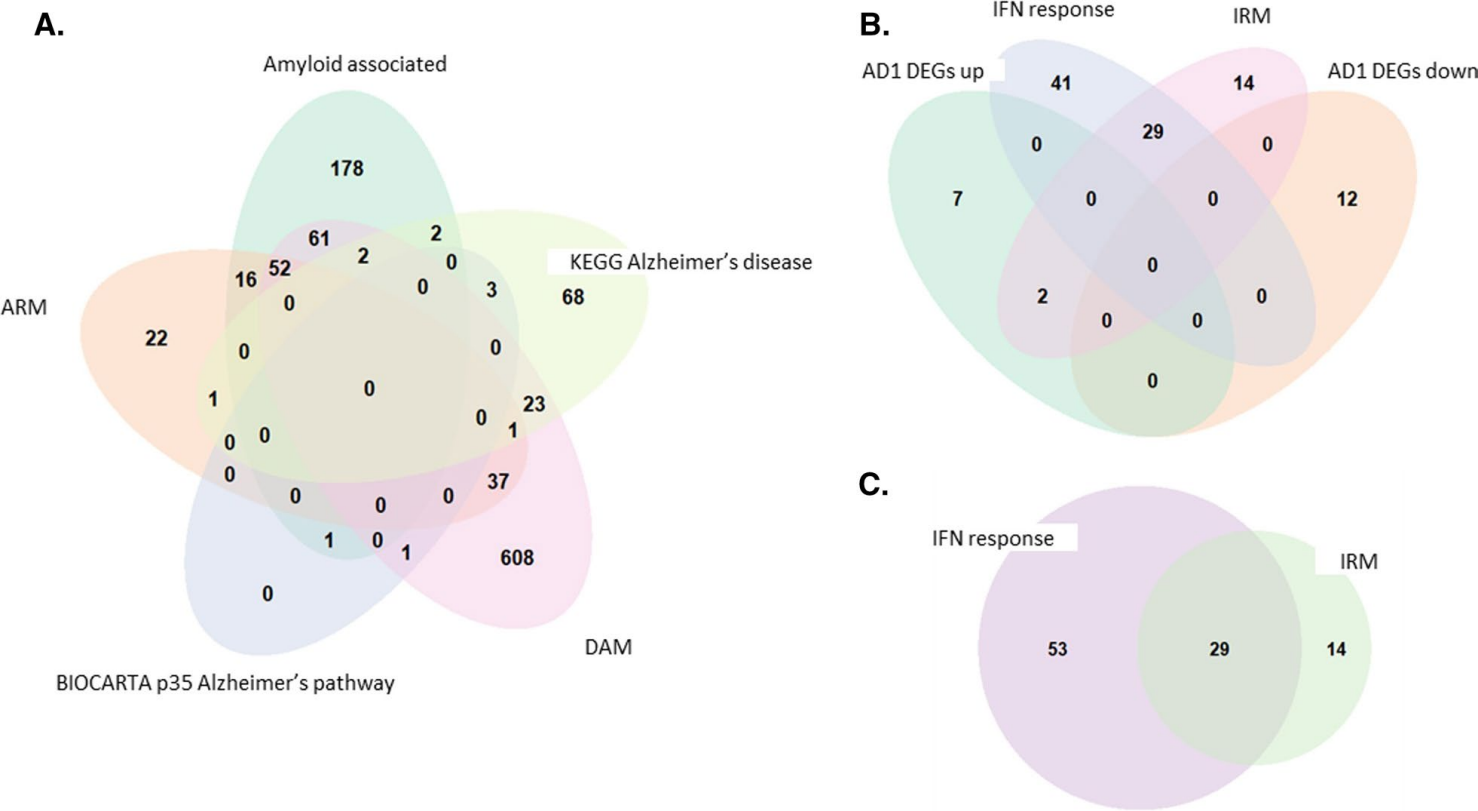
Overlapping leading edge genes for the female-enriched Alzheimer’s disease associated gene sets (AD-GS). Venn diagrams showing the overlapping and unique leading-edge genes for each of the IR-AD found significantly enriched in the myeloid cells isolated from women in the **A** MDMs, **B** MCs, and **C** MGLs

We next sought to determine whether the sex differences in the observed enrichments were driven by a small subset of genes shared between the gene sets. These ‘driver’ genes are known as leading-edge genes as they account most for the enrichment of a specific gene set. Accordingly, leading edge analysis was performed on the female enriched IR-AD gene sets from each of the peripheral myeloid population analysed via GSVA (non E2-treated F-MCs, F_lo_-MDMs, and F-MGL). Overlapping leading-edge genes were identified for the enriched IR-AD gene sets in each population, although, a substantial number also were identified that were unique to each of the gene sets analysed (Fig. 4A–C). This suggests that the enrichment of IR-AD gene sets in cells derived from women is driven by an upregulation of multiple genes whose differential expression may be associated with AD, rather than a small subset.

Finally, many of the IR-AD gene sets tested were first defined in mouse models of AD. To confirm that the genes driving the enrichment of mouse microglial signatures in female cells also are associated with human microglia, we tested whether the leading-edge genes were over-represented in previously identified human microglial gene co-expression modules. These modules were previously identified via co-expression network analyses to characterise potential functional relationships in microglia, described in Smith et al*.* [39]*,* and were selected based on the correlation of their average expression in microglial nuclei with amyloid beta and pTau in the tissue from which they were isolated. Notably, the leading edge genes were found to be significantly over-represented in the AD-related gene co-expression modules 2, 19, and 24 all of which significantly correlated with pTau expression. Module 2 was enriched in the amyloid-associated, ARM, and DAM leading edge genes (*p* < 0.0001 for all) for the F_lo_-MDMs, whilst DAM additionally exhibited a significant association with module 19 (*p* < 0.0001). Furthermore, leading-edge genes driving the IRM gene set in the aged MCs displayed significant overlap with module 19 (*p* = 0.037), which included expression of APOE as a hub gene, whilst both IFN response and IRM leading edge genes in the MCs and MGL significantly overlapped with module 24 (*p* < 0.0001 for all). Taken together, these data provide further evidence suggesting that the enrichment of IR-AD gene sets in myeloid cells from women are relevant to AD pathology.

## Discussion

Sex differences in prevalence can be observed across the spectrum of diseases associated with dysregulated immune responses [2]. This includes AD, for which genetic evidence suggests that microglia may play a causal role [12–15]. Here, we sought to determine whether microglia isolated from women with no ante-mortem neurological disease exhibit a transcriptional signature that is biased towards pathways relevant to AD relative to those isolated from men. snRNA-seq data previously generated by our laboratory [39] revealed that genes associated with an increased risk of AD were significantly enriched in microglial nuclei isolated from brains of women without neurological disease relative to those from men. This finding was replicated in a larger, independent brain microglial snRNA-seq data set [41]. Further analysis determined that brain microglial gene signatures in women were enriched in gene sets whose expression was associated with the accumulation of pathological human β-amyloid or phosphorylated tau species in transgenic mouse models [31, 43–46]. Genes significantly upregulated in microglia from women were primarily associated with a pro-inflammatory immune response. A consistent pattern was observed in similar analyses of peripheral myeloid cell populations (MCs and MDMs). The relative gene set enrichment in peripheral myeloid cells was not modulated by short-term exposure to oestrogen in vitro. These data are consistent with the hypothesis that the increased risk of AD in women may be partly explained by a sex-specific microglial phenotype which is transcriptionally predisposed to processes relevant to the disease pathology. A notable exception to the consistent results found throughout the data sets analysed here is the lack of enrichment of inflammatory and IR-AD gene sets in the microglial nuclei isolated from individuals with AD. This suggests that the sex differences observed in the non-disease populations are reflective of an increased baseline expression of genes relevant to AD pathology in women, potentially predisposing them towards the disease pathology, i.e., it is a factor contributing to susceptibility. This hypothesis is consistent with loss of this difference in AD patients; whilst susceptibility to the disease may be increased, the expression of the disease once established is not changed.

Previous studies of sex differences in the human peripheral blood transcriptome exhibit inconsistent results [52, 53], but these data may have been confounded by differences in frequencies of immune cell populations between the sexes [54]. Those studies assessing transcriptional profiles of isolated monocytes alone reported evidence for sex-specific effects consistent with our findings. For example, the transcriptome of peripheral monocytes isolated from individuals with evidence for chronic-low level inflammation found augmented expression of pro-inflammatory genes in women relative to men [55]. Moreover, in patients diagnosed with early Parkinson’s disease, inflammatory activation of monocytes was observed in cells from women, but not men [56]. These studies also provided evidence for enrichment of genes associated with the IFN-response in peripheral monocytes from women, as we report here [52, 55, 56]. Whilst we observe clear sex differences in the non-diseased monocytes, we did not find a data set that allows us to determine whether these differences were retained in monocytes isolated from individuals with AD.

Sex differences have also previously been reported in the human microglial transcriptome. Using computational cell mapping techniques to predict microglial gene expression from bulk transcriptomic data, Bonham et al. [33] inferred dysregulation of microglial genes in AD patients in the cerebellum and temporal cortex, and these perturbations were observed to a greater extent in women compared to men. Moreover, using snRNA-seq Mathys et al. [57] identified a subcluster of AD-associated microglia enriched in the brains of women relative to men. However, due to the relatively rarity of microglia in the brain, in this un-enriched population only a low resolution of microglia was be obtained (*n* = 1920 microglial nuclei compared to *n* = 27,592 in our enriched discovery data set [39] and *n* = 148,606 in the replication data set [41]). However, despite the large number of nuclei, these data sets were derived from a small number of individuals. Additionally, considering APOE is the main genetic risk factor for AD, it would be of interest to confirm these results in a larger sample size in which the APOE genotype is known. Nevertheless, the results from these studies support the hypothesis that the female bias observed in AD diagnosis may be partially driven by sex differences in microglial phenotypes.

The lack of evidence in our study for an E2-mediated effect on the GWAS and AD related gene sets analysed suggests that the sex differences we observed arise as a consequence of differential regulation of expression of genes on the sex chromosomes. The X chromosome includes the largest number of annotated immune related genes in the human genome [2], including the X-linked helicase *DDX3X*, an upstream regulator of type I interferon [58]; the toll-like receptors, *TLR7* and *TLR8* [59]; and *IRAK1*, *NEMO*, *NKRF*, and *NKAP*—members of the NF-κB signalling pathway [59]. Moreover, genes which have been shown to escape X-chromosomal inactivation, including *TLR7* and *IRAK1* [38], have the potential to functionally influence phenotypic diversity between male and female immune cells. Future research could study this directly by exploring sex-specific enhancer-promoter interactions for the sex chromosomes in microglia.

Although we have made convergent observations using different myeloid cell data sets generated both in house and externally, our study has several limitations. We have based our rationale on an interpretation of the predominant AD associated gene expression enrichment for microglia in the context of cells in the brain yet relied in part on supportive evidence from data concerning peripheral monocytes and macrophages. This genomic evidence could be interpreted more broadly as implicating sex-specific myeloid (as well as yolk sac derived microglia) cells in determining the relatively higher frequency of AD amongst women. Moreover, as the monocyte data set was not designed to study AD, information on their amyloid and tau burden was not available. However, as the median age was 67, it is likely that only around 0.6% of individuals had any form of dementia [60] which would be too small to account for the transcriptomic differences we report. Moreover, whilst amyloid deposits have been found in the brains of ~ 22% of cognitively normal individuals, no significant sex differences in total amyloid burden were reported [61, 62] and thus this is unlikely to account for the transcriptional enrichments observed here. Furthermore, due to the difficulties in obtaining post-mortem microglia from younger donors, the age-independence of the sex differences in myeloid cells transcriptome was based on the analysis of peripheral populations. Berchtold et al., has previously identified sexually dimorphic changes in gene expression across the brain as a consequence of aging [63]. Thus, we cannot rule out that there may be an interaction between the effects we describe and the age of the donors.

Relevant in vivo differences in monocytes across the menstrual cycle may have been lost as a consequence of the 7-day in vitro MDM culture and our study of the effects of E2 treatment used only short treatment periods. Moreover, due to practical considerations only a small number of male and female iPSC lines were used. Nevertheless, our conclusion that the observed expression enrichments are independent of sex hormones is supported by the fact that these enrichments were detected in both monocytes and post-mortem microglia from post-menopausal women. It is important to note, however, that our determination of a post-menopausal state was based on age alone and thus the donors may not all have been at the same menopausal stage.

Finally, all of our work is based on transcriptomic evidence because of its broad coverage and the confidence with which it can be related to genetic association study data. Similar interrogations of expressed proteins are needed as comparable cell-specific untargeted proteomic data of sufficient size becomes available.

In conclusion, we present transcriptomic data from multiple human-derived myeloid populations supporting the hypothesis that the increased risk of AD in women may be partly explained by a sex-specific microglial/myeloid phenotype which is biased towards processes involved in the disease pathology. Our identification of molecular evidence for sex-specific susceptibility effects for AD suggests that future research into mechanisms for sex-associated gene regulation may provide novel targets for modification of disease susceptibility.

## Materials and methods

### Cell culture

#### Generation and culture of monocyte-derived macrophages

##### Donors

Written informed consent was obtained from each donor prior to the collection of blood (Hammersmith Hospital, London, England) in accordance with Research Ethics Committees reference number 12/LO/0538. Subject inclusion criteria were premenopausal women (*n* = 10, age = 26.3 ± 3.9) and age-matched males (*n* = 7, age = 26.8 ± 4.4). Subjects were excluded if they were currently using medications known to interfere with immune or hormonal function, or if they had a diagnosis of any chronic inflammatory condition. Women also were excluded if they were pregnant or breastfeeding, had any known diagnosis of polycystic ovary syndrome, had any periods of amenorrhea in the past 6 months, or were using medications known to interfere with the menstrual cycle. Blood was collected from women on two occasions: once during menstruation (F_lo_), and once during the late follicular phase of the menstrual cycle (F_hi_), determined via subtracting 16 days from the predicted start date of their consecutive cycle. Aliquots of serum were collected from each participant and total E2 quantified via the Abbott Alinity platform, analysed by the Charing Cross Hospital Biochemistry laboratory (London, England).

### Cell culture

Peripheral monocytic cells were isolated from the buffy coats of freshly collected blood via gradient separation (Histopaque 1077, Sigma-Aldrich) and purified by magnetic activated cell sorting using CD14+ MicroBeads and LS columns (Miltenyi Biotech). The CD14^+^ cells were resuspended in phenol red free RPMI 1640 medium (Gibco) supplemented with 10% charcoal-stripped FBS (Foetal Bovine Serum; Gibco) and 25 ng/mL M-CSF (Gibco). After 3 days of culture at 37 °C with 5% CO_2_, non-adherent cells were aspirated, and those remaining were given fresh medium containing 50 ng/mL M-CSF. Following 7 days of differentiation, cells were given a complete medium change. After 6 h, cells were harvested for RNA sequencing, by lysing cells in 500 µL QIAzol Lysis Reagent (QIAGEN) for 2–3 min, and stored at − 80 °C.

### Generation of pluripotent stem cell-derived microglial-like cells

#### Differentiation and culture of microglial-like cells

Three female (WTSIi003, WTSIi040, WTSIi087) and three male (WTSIi023, WTSIi090, WTSIi107) human iPSC lines were purchased from the European Collection of Authenticated Cell Cultures (EACACC) and cryopreserved in liquid nitrogen. Upon thawing, iPSCs were seeded onto Geltrex (Gibco) coated plates and cultured in OxE8 medium [64] at 37 °C with 5% CO_2_. For the initial 24 h after thawing the cells, OxE8 medium was supplemented with 10 μm/mL ROCKi (Rho kinase inhibitor; abcam). At a confluency of approximately 70%, iPSCs were split using 0.5 mM EDTA (Ethylenediaminetetraacetic acid; Invitrogen) in PBS. The production of iPSC-derived myeloid precursor cells was performed in accordance with the protocol outlined by van Wilgenburg et al. [65], with a few changes as detailed in Additional file 1. Harvested myeloid precursor cells were plated for differentiation to microglial-like cells (MGL) over the course of 7 days in accordance with the protocol outlined by Haenseler et al. [51]. To mitigate the variability induced via the culturing process, this was repeated twice for each cell line and the samples were pooled prior to analysis. To confirm the successful generation of an MGL phenotype, the expression of a set of 6 microglial genes [66] was examined in their respective bulk RNA-seq data set: *P2RY12*, *TMEM119*, *IBA1*, *ITGAM*, *ITGAX* and *CX3CR1*. Across both sexes, all cells expressed these microglial genes (Additional file 1: Fig. S4).

### Transcriptomic data analysis

#### Pre-processing

##### Isolation, sequencing, and pre-processing of microglia nuclei

Single nuclei were previously isolated within our laboratory from fresh frozen entorhinal and somatosensory cortical tissue blocks of neocortical grey matter of non-diseased control male (*n* = 6600 nuclei from 4 donors; aged 77 ± 3.58) and female (*n* = 4634 from 2 donors; aged 85.5 ± 6.35) brains, and from AD male (*n* = 8,086 from 3 donors; aged 82.4 ± 2.76) and female brains (*n* = 3,959 from 2 donors; aged 76.5 ± 9.81), as previously described [39], for further clinical details see Additional file 2: Table S8. In addition, we re-analysed data from nuclei isolated from control male (*n* = 34,027 from 10 donors; aged 70.6 ± 7.17) and female (*n* = 24,909 from 6 donors; aged 72 ± 5.87) brains alongside AD male (*n* = 17,891 from 4 donors; aged 77 ± 2.31) and female (*n* = 91,859 from 16 donors; aged 76.9 ± 2.5) brains generated via Gerrits et al. [40]. The two data sets were jointly analysed exactly as previously described [67] using default parameters of the scFlow pipeline [68].

##### Filtering the population of monocytes from the E-GEOD-56047 data set

The ArrayExpress Archive of Functional Genomics Data was searched for RNA sequencing data on human monocytes obtained from non-diseased, post-menopausal, age- and sex-matched donors. The microarray data set E-GEOD-56047 was selected for analysis as it contained transcriptomic information on monocytes isolated from a total of 1202 men and women ranging from 44 to 83 years of age. The data set was first filtered to include only CD14^+^ cells and, as no sample-specific sex information was contained within the metadata, expression of the Y-linked gene *RPS4Y1* was used to sex the samples. Plotting the *RPS4Y1* count data revealed two clear peaks of expression: one, centring around a count value of 20, came from the female-derived samples, whilst the other, centring around a count value of 5650, was assumed to contain the males (Additional file 1: Fig. S1A). To minimise the possibility of misclassification, a stringent maximum threshold of 200 counts was set for samples to be classified as female-derived, whilst a minimum threshold of 3750 was set for samples to be classed as males. 95 samples whose *RPS4Y1* counts fell between the two were removed from further analysis. Moreover, as more than 99% of women experience menopause at the age of 59 [69], only samples from women older than 59 were included in the analysis, allowing analyses of 296 post-menopausal monocytes from women (F-MCs) and 297 age-matched monocytes from men (M-MCs) (Additional file 1: Fig. S1B).

##### RNA extraction, sequencing and processing of bulk RNA sequencing (RNA-seq)

RNA was extracted using QIAzol Lysis Reagent the RNeasy Mini Kit (QIAGEN) with optional on-membrane DNase digestion performed using 10 µL DNase I stock solution (QIAGEN) in accordance with manufacturer’s instructions. The eluted RNA was stored at − 80 °C. For the MDMs, only samples with > 100 ng total RNA were used for downstream sequencing (*n* = 24, comprised of 7 male, 10 F_lo_ and 7 F_hi_ donors), with these samples displaying RIN values of 9.54 ± 0.46. In contrast, for the MGLs, 200 ng of total of RNA was used as input for cDNA library construction. Detailed information on the sequencing and processing of the bulk RNA can be found in the appendix.

### Statistical analysis

#### Enrichment of GWAS loci in post-mortem microglia

GWAS summary statistics for AD [14] were tested for enrichment in microglial nuclei and monocytes samples using MAGMA (v1.08) [70] and MungeSumstats (v1.1.24) [71] packages [40]. First, summary statistics were mapped to genes using the map.snps.to.genes function of MAGMA.Celltyping (v1.0.1) [40]. The enrichment of microglia from single nucleus RNA sequencing (snRNA-seq) studies was performed as follows: First, the normalized expression matrix was extracted for each nucleus. Considering snRNA-seq data show a considerable number of dropouts (i.e., zero values in the expression matrix of each nucleus, notably because the starting material for the sequencing from each individual nucleus is particularly low), the number of distinct features identified in each nucleus varied across the data set. A higher number of distinct features (or a lower number of dropouts) identified in a nucleus might bias the results of the MAGMA enrichment analysis towards a higher value for this particular nucleus and vice versa. To reduce the effect of dropouts on the calculation of nuclear enrichment, only the top *n* features per nucleus were included in the calculation of MAGMA enrichment, where *n* is the 75th percentile of the number of distinct features per nucleus across the nuclei of the snRNA-seq data set. MAGMA was then run with the default parameters, using a positive-direction = pos and the expression matrix as a covariate. The resulting MAGMA enrichment *P* values for the microglial nuclei and the monocyte samples were compared between those collected from female and those collected from male donors. Regarding the snRNAseq microglial results, the lme4 package in R was employed using the following model in the lmer function: sqrt(− log10P) ~ Sex + Sex:Disease + (1|Sample). Here, donors’ sex is the independent variable, a possible disease status-specific effect of sex is assessed using the interaction term, and the tissue sample is included as a random effect variable (to control for the effect of pseudoreplication bias). In a similar way, normalized gene expression values in the monocyte data set were used. As this was bulk RNAseq data with minimal drop out compared to snRNAseq, the expression values from the whole transcriptome were used as input. To determine significance, a Wilcoxon rank sum test was employed in R.

#### Differential gene expression analysis in single nuclei RNA sequencing (snRNA-seq) data

MAST analysis [42] was used to identify any differentially expressed genes between male and female microglial nuclei in non-disease control and AD samples separately via a zero-inflated mixed model analysis. This was performed using a downloadable in-house developed script available from the R package scFlow [68]. The model specification was as follows: zlm(~ sex + (1|sample) + cngeneson + pc_mito + brain_region + age, method = “glmer”, ebayes = F). Each nuclei preparation is used as a random effect (sample), and sex, the cellular detection rate (cngeneson), the percentage of counts mapping to mitochondrial genes (pc_mito), the brain region and age are included as fixed effects in the model. Only genes expressed in at least 10% of nuclei were included in the analysis. Unless stated otherwise, genes with a false discovery rate corrected *p* value of < 0.05 were deemed significant. Enrichment analyses of GO biological processes were performed on the significant DEGs using the R package enrichR [72]. Where many pathways came up as enriched, an in-house developed script [68] was used to cluster the functionally related groups based on a similarity score generated via Cohen’s kappa statistics on the overlapping genes between the enriched pathways following the original algorithm used in the DAVID gene functional classification tool [73]. The R package AUCell (v 1.6.1)[74] was used to determine the enrichment of the IR-AD (inflammatory response in AD) gene sets; their respective genes can be seen in Additional file 2: Table S2. This generated scores analogous to the gene set enrichment scores determined via GSVA, as described later. To compare the enrichment of the gene sets between nuclei from male and female subjects, a linear model was fit to the AUCell scores matrix using the function lmFit with sex, the number of features detected, the percentage of counts mapping to mitochondrial genes, and the brain region included as fixed effects. Pseudo-replication bias was corrected using the duplicateCorrelation function of the limma R package [75] with sample as the “blocking” variable. Finally, the function eBayes was employed to compute moderate *t*-statistics and perform multiple test corrections. Unless stated otherwise, pathways with a false discovery rate < 0.05 were considered significantly differentially enriched.

#### Differential gene expression analysis of monocytes from the E-GEOD-56047 data set

Data was analysed using the R package limma [75]. First, the data was normalised using the function rma, and a simple linear model was built with sex only as the fixed effect. Finally, for statistical analysis and assessing differential expression, the empirical Bayes method was employed using the function eBayes and multiple testing was performed via the Benjamin–Hochberg procedure. Unless stated otherwise, genes with a false discovery rate corrected *p* value of < 0.05 were deemed significant.

#### Differential gene expression analysis of MDMs

Prior to analysis, pseudogenes—identified via the R package biomaRt [76]—and genes with a low expression count, < 10 reads across all samples, were removed from the raw expression data. Gene counts were then normalised using the DESeq2 R package [77]. For unpaired analysis the model matrix was designed using group (M, F_lo_, and F_hi_) and treatment only. For the paired analysis (F_lo_ vs F_hi_), subject ID was included as a nested factor. Differential gene expression analyses were performed on the normalised count matrix in a pairwise manner using the DESeq2 package. Default parameters were employed throughout DEseq2 usage, including the Wald test of significance. Multiple testing was performed via the Benjamin–Hochberg procedure. Unless stated otherwise, genes with a false discovery rate corrected *p* value of < 0.05 were deemed significant.

#### Gene set variation analyses of MDMs, MGL, and monocytes

GSVA were performed on the filtered raw expression matrix using the R package GSVA [49]. The R package Biobase [78] was used to extract the relevant canonical pathways from the C2 collection of the Molecular Signatures Database (MSigDB) gene sets (Additional file 2: Table S9). The GSVA function inside the GSVA R package was used to generate gene set enrichment scores and differential expression was then examined using the R package limma. First, a linear model was fit to the gene set enrichment score matrix using the function lmFit, and subsequently the function eBayes was employed to compute moderate *t*-statistics and multiple test corrections. Unless stated otherwise, pathways with a false discovery rate of less than 0.05 were considered enriched and the adjusted *p* values reported. Finally leading-edge analyses were performed on the female enriched IR-AD using the ranked gene list generated via the GSVA package, using the compute_rank_score function. These scores were averaged across the respective female samples and leading-edge genes were determined using the fgsea package [79].

## Supplementary Information

**Additional file 1: Figure S1.** Expression of microglial genes in MGLs. Violin and box plots of the normalized expression counts of key microglial genes, including *IBA1*/*AIF1*, *ITGAM* (CD11b), *ITGAX* (CD11c), *P2RY12*, *TMEM119*, and *CX3CR1* in male and female induced pluripotent stem cell-derived microglial-like cells (MGLs). **Figure S2.** Sex labelling and identification of a population of post-menopausal female and age-matched male monocytes. **A** Histogram showing expression counts of the Y-chromosomal gene, *RPS4Y1*, in a collection of 1202 monocyte samples derived taken from the publicly available microarray data set E-GEOD-56047. To sex the samples, expression counts of *RPS4Y1* which fell below 200 were classed as female whilst expression counts exceeding 3,750 were classed as male. Samples whose expression fell between these values were left as unknowns. **B** Box and whisker plot showing the age of MCs following exclusion of all samples younger than 59 years of age, *n* = 297 male, 296 female, and 56 unknown. **Figure S3.** Serum concentrations of E2. Box plot showing differences in the serum concentrations of 17β-estradiol (E2). Significance was determined via an unpaired *t* test for the male–female comparisons and a paired *t* test to compare across the menstrual cycle. Samples whose serum E2 fell below 100 pg/mL were not able to be quantified and thus they were excluded from the analysis. FDR corrected *p* values are shown. (*p < 0.05; **p < 0.01). **Figure S4.** Oestrogen receptor expression in MGLs. Violin and box plots showing the kernel probability, the median, and interquartile range of the normalized expression counts of *ESR1* and *GPER1*.

**Additional file 2: Table S1.** Sexually differentially expressed genes in the microglial nuclei. **Table S2.** Genes comprising each of the IR-AD gene sets. **Table S3.** Sexually differentially expressed genes between the F- and M-MCs. **Table S4.** GSVA of IR-AD gene sets between F_lo_- and F_hi_-MDMs results. **Table S5.** Sexually differentially expressed genes between the F_lo_- and M-MDMs. **Table S6.** Sexually differentially expressed reactome pathways between the F_lo_- and M-MDMs. **Table S7.** Differentially expressed reactome pathways between the F_lo_- and F_hi_-MDMs. **Table S8.** Smith et al. [39], sample specific metadata. **Table S9.** Reactome gene sets used for the analysis of the MDMs.

## Data Availability

The data that support the findings of this study are available from the corresponding author, including all in-house developed scripts, upon request.

## Abbreviations

AD: Alzheimer’s disease
E2: 17β-Oestradiol
EWAS: Epigenome wide association study
F-: Female
F_hi_: Samples isolated from women during the predicted late follicular stage of their menstrual cycle
F_lo_: Samples isolated from women during menstruation
GO: Gene ontology
GSVA: Gene set variation analysis
GWAS: Genome wide association study
IR-AD: Inflammatory response in Alzheimer’s disease
M-: Male
MC: Male monocyte
MDM: Monocyte derived macrophages
MGL: Microglial-like cell
MGN: Microglial nuclei
sDEG: Sexually differentially expressed genes
sDEGS: Sexually differentially expressed gene set
