# Supplementary material for: Alzheimer’s disease-related transcriptional sex differences in myeloid cells
Source: J Neuroinflammation. 2022 Oct 5;19:247. doi: 10.1186/s12974-022-02604-w (PMC9535846; doi:10.1186/s12974-022-02604-w)
Supplement: Supplementary file 1 — Additional file 1: Figure S1. Expression of microglial genes in MGLs. Violin and box plots of the normalized expression counts of key microglial genes, including IBA1/AIF1, ITGAM (CD11b), ITGAX (CD11c), P2RY12, TMEM119, and CX3CR1 in male and female induced pluripotent stem cell-derived microglial-like cells (MGLs). Figure S2. Sex labelling and identification of a population of post-menopausal female and age-matched male monocytes. A Histogram showing expression counts of the Y-chromosomal gene, RPS4Y1, in a collection of 1202 monocyte samples derived taken from the publicly available microarray data set E-GEOD-56047. To sex the samples, expression counts of RPS4Y1 which fell below 200 were classed as female whilst expression counts exceeding 3,750 were classed as male. Samples whose expression fell between these values were left as unknowns. B Box and whisker plot showing the age of MCs following exclusion of all samples younger than 59 years of age, n = 297 male, 296 female, and 56 unknown. Figure S3. Serum concentrations of E2. Box plot showing differences in the serum concentrations of 17β-estradiol (E2). Significance was determined via an unpaired t test for the male–female comparisons and a paired t test to compare across the menstrual cycle. Samples whose serum E2 fell below 100 pg/mL were not able to be quantified and thus they were excluded from the analysis. FDR corrected p values are shown. (*p < 0.05; **p < 0.01). Figure S4. Oestrogen receptor expression in MGLs. Violin and box plots showing the kernel probability, the median, and interquartile range of the normalized expression counts of ESR1 and GPER1. [file 12974_2022_2604_MOESM1_ESM.docx]

**Additional file for**

Alzheimer’s disease related transcriptional sex differences in myeloid cells.

Isabelle Coales, Stergios Tsartsalis, Nurun Fancy, Maria Weinert, Daniel Clode, David Owen, Paul M. Matthews

**Correspondence to:**

Prof. Paul Matthews, E502, Burlington Danes Building, Hammersmith Hospital, DuCane Road, London W12 0NN UK. [p.matthews@imperial.ac.uk](mailto:p.matthews@imperial.ac.uk)

or

Dr. David Owen, Clinical Research Facility, ICTM Building, Hammersmith Hospital, DuCane Road, London W12 0NN UK. [d.owen@imperial.ac.uk](mailto:d.owen@imperial.ac.uk)

**This PDF file includes:**

Detailed Methods

Figures S1 to S4

Titles for Tables S1 to S9

**Other additional materials for this manuscript include the following:**

Tables S1 to S9

**Generation of pluripotent stem cell-derived microglial-like cells**

**Generation of myeloid precursor cells from iPSCs:** The production of iPSC-derived myeloid precursor cells was performed in accordance with the protocol outlined by van Wilgenburg et al., (2013) [1], with a few changes as detailed: To generate embryoid bodies (EBs), adherent iPSCs were detached by incubating the cells in 450 µL TrypLE Express (Gibco) for 3 mins at 37°C. Cells were subsequently pipetted to single cell suspension and diluted 1∶10 with PBS. Following centrifugation at 600 g for 5 mins, cells were resuspended at 4x106 cells/mL in EB medium (OxE8 medium supplemented with 50 ng/mL BMP4 (PeproTech), 50 ng/mL VEGF (PeproTech) and 20 ng/mL SCF (Miltenyi Biotec)), supplemented with 10 μL/mL ROCKi, seeded into an AggreWell™ 800 plate (Stemcell Technologies) (pre-rinsed with 500 μL AggreWell rinsing solution (Stemcell Technologies) and washed with 1.5 mL KO-DMEM) and centrifuged at 300 g for 2 min, with low acceleration and deceleration. The iPSCs were then given daily feeds of fresh EB medium for 7 days. At day 7, the EBs transferred to an ultra-low adherence plate (Corning) in FM7a medium (Advanced DMEM/F12 (Gibco) supplemented with 20 mM GlutaMAXTM (Gibco), 15mM HEPES (Gibco), 250 µL stabilised insulin (Sigma-Aldrich), 15 µM Tropolone (Sigma-Aldrich), 25 ng/mL IL-3 (PeproTech) and 100 ng/mL M-CSF). The EBs were then transferred to two T175 flasks containing 20 mL FM7a medium each. Once weekly, 10 mL FM7a medium was added to the flask until myeloid precursor cells were visible in the culture medium; the non-adherent cells were then harvested weekly via collection of 25 mL supernatant from each factory, replaced by an equal volume of fresh FM7a medium.

Differentiation and culture of microglial-like cells: The myeloid precursor cells were plated into 6-well plates for differentiation to microglial-like cells (MGL). Here, factory supernatant was collected in 50 mL falcon tubes and spun at 600 g for 7 mins and the cell pellet resuspended at 1.5x10^5^ cells/mL in MIC10 medium (SILAC Advanced DMEM/F12 (Gibco) supplemented with 20 mM GlutaMAX^TM^, 10 mM glucose (Sigma-Aldrich), 0.5 mM L-lysine (Fisher Scientific), 0.7 mM L-arginine (Fisher Scientific), 100 ng/mL IL-34 (Invitrogen) and 10 ng/mL GM-CSF) for microglial differentiation. Cells then were seeded at a density of 1.5x10^5^ cells/well. After 3 days in culture, cells were fed with fresh MIC10 medium. Following 7 days of differentiation, half of the plated MGL for each cell line were treated with fresh MIC10 medium supplemented with 13 pmol/L [2] (representing 1.3 % free of a total circulating 1000 pmol/L) of 17β-estradiol (E2, Sigma-Aldrich) for 24 hrs. To obtain a stock concentration of 0.1 mg/mL E2, 1 mg powdered E2 was dissolved in 10 mL absolute ethanol and passed through a WhatmanTM 0.2 µm syringe filter prior to further dilution in MIC10 medium. Parallel wells treated with an equivalent final concentration of EtOH (E2 controls).

Sequencing and data processing of monocyte-derived macrophages (MDMs): Samples were sent to Genewiz (Hertfordshire, England) where RNA was quantified via absorption (Qubit) and RNA integrity number (RIN) assessed using Tapestation (Agilent). Total RNA concentrations ranged from 16.2 ng to 1517.88 ng, with a mean value of 482.75 ng. In order to include as many samples as possible in the analyses, only samples with > 100 ng total RNA were used for library construction via QuantSeq 3’ mRNA-Seq Library Prep Kit FWD for Illumina (Lexogen). Libraries were run in single index on HiSeq (llumina) in 2 x 150 bp configuration per lane to obtain an average read depth of ~ 10M reads per sample. Data processing was performed via nf-core-rnaseq (V 1.4.2) [3] pipeline. Firstly, Trim Galore! (V 0.6.4) [4] was employed to trim the adapter sequence ('AGATCGGAAGAGC'), with a maximum trimming error rate of 0.1 (default), a minimum required adapter overlap of 1 bp, and a minimum required sequence length of 20 bp. To ensure the removal of low-quality reads from samples a quality phred score cut-off of 20 was used and sequences were trimmed by 18 bp from their 3' end to avoid poor qualities or biases. Processed reads were mapped to the GRCh38 human reference genome via STAR (v 2.7.0a) [5] using gene annotations from Ensembl (v 97). For STAR mapping default settings were employed with the addition of (i) --twopassMode set to Basic and (ii) --quantMode set to GeneCounts for gene-level abundance estimates.

Sequencing of microglial-like cells RNA and data pre-processing: Sample QC, library preparation and sequencing were performed by the Imperial BRC Genomics facility (London, England). RNA was quantified and RNA integrity number (RIN) assessed using Tapestation (Agilent). Total RNA concentrations ranged from 203.4 ng to 1377 ng, with a mean value of 613.6 ng. Library construction was performed on 200 ng total RNA via QuantSeq 3’ mRNA-Seq Library Prep Kit FWD for Illumina (Lexogen). Libraries were run in single index on HiSeq 400 75 PE (lllumina) in 2 x 150 bp configuration per lane to obtain an average read depth of ~ 10M reads per sample. Raw data files were processed for fastq by the Imperial BRC Genomics Facility using the software Bcl2Fastq, this pipeline included demultiplexing and the trimming of Illumina generic adapters from the reads with the setting set to default. Subsequent data processing was performed via nf-core-rnaseq (V 1.4.2) [3]. Processed reads were mapped to the GRCh38 human reference genome via STAR (v 2.7.0a) [5] using gene annotations from Ensembl (v 97), as described above.


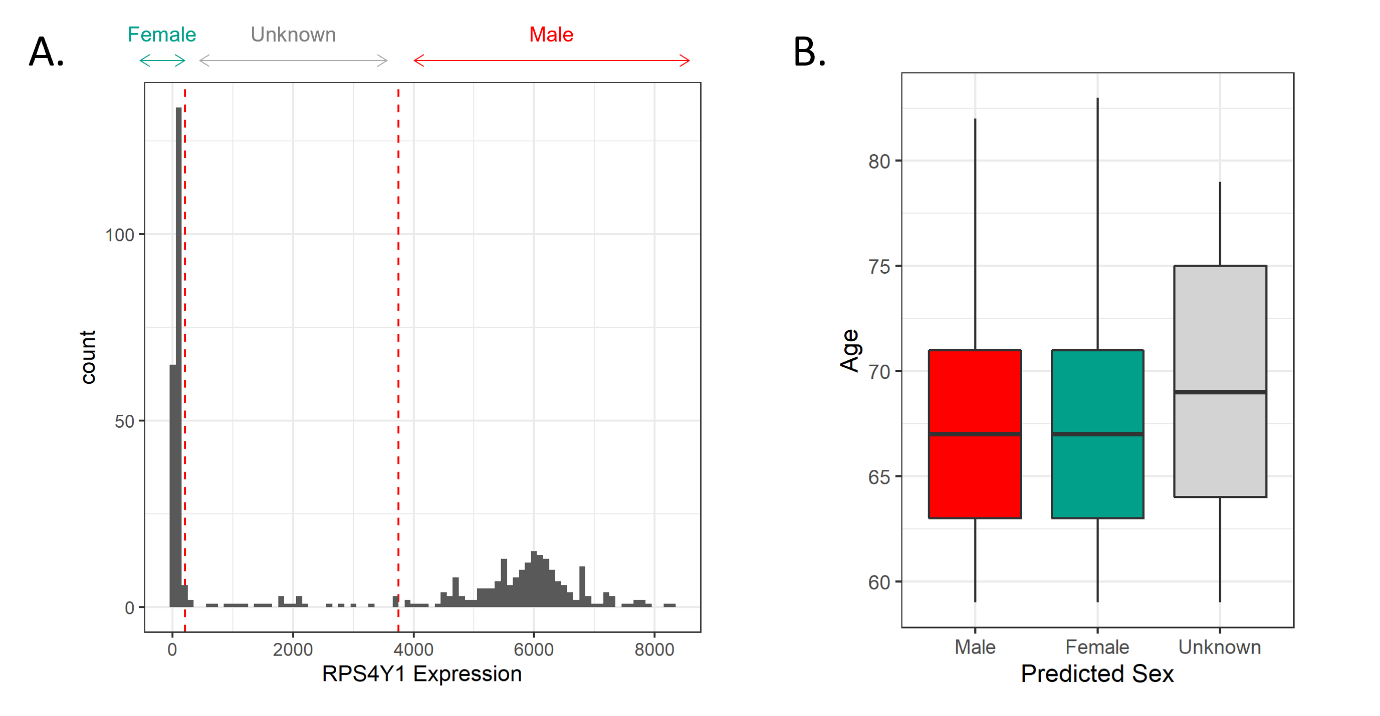


Fig. S1. Sex labelling and identification of a population of post-menopausal female and age-matched male monocytes: (A) Histogram showing expression counts of the Y-chromosomal gene, RPS4Y1, in a collection of 1,202 monocyte samples derived taken from the publicly available microarray dataset E-GEOD-56047. To sex the samples, expression counts of RPS4Y1 which fell below 200 were classed as female whilst expression counts exceeding 3,750 were classed as male. Samples whose expression fell between these values were left as unknowns. (B) Box and whisker plot showing the age of MCs following exclusion of all samples younger than 59 years of age, n = 297 male, 296 female, and 56 unknown.


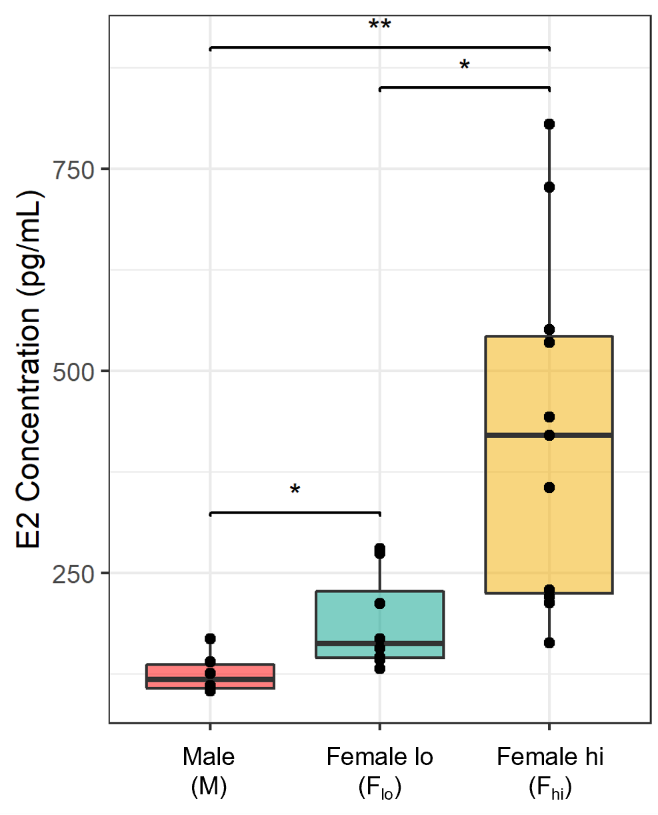


Fig. S2. Serum concentrations of E2: Box plot showing differences in the serum concentrations of 17β-estradiol (E2). Significance was determined via an unpaired t-test for the male-female comparisons and a paired t-test to compare across the menstrual cycle. FDR corrected p-values are shown. (* p < 0.05; ** p < 0.01).


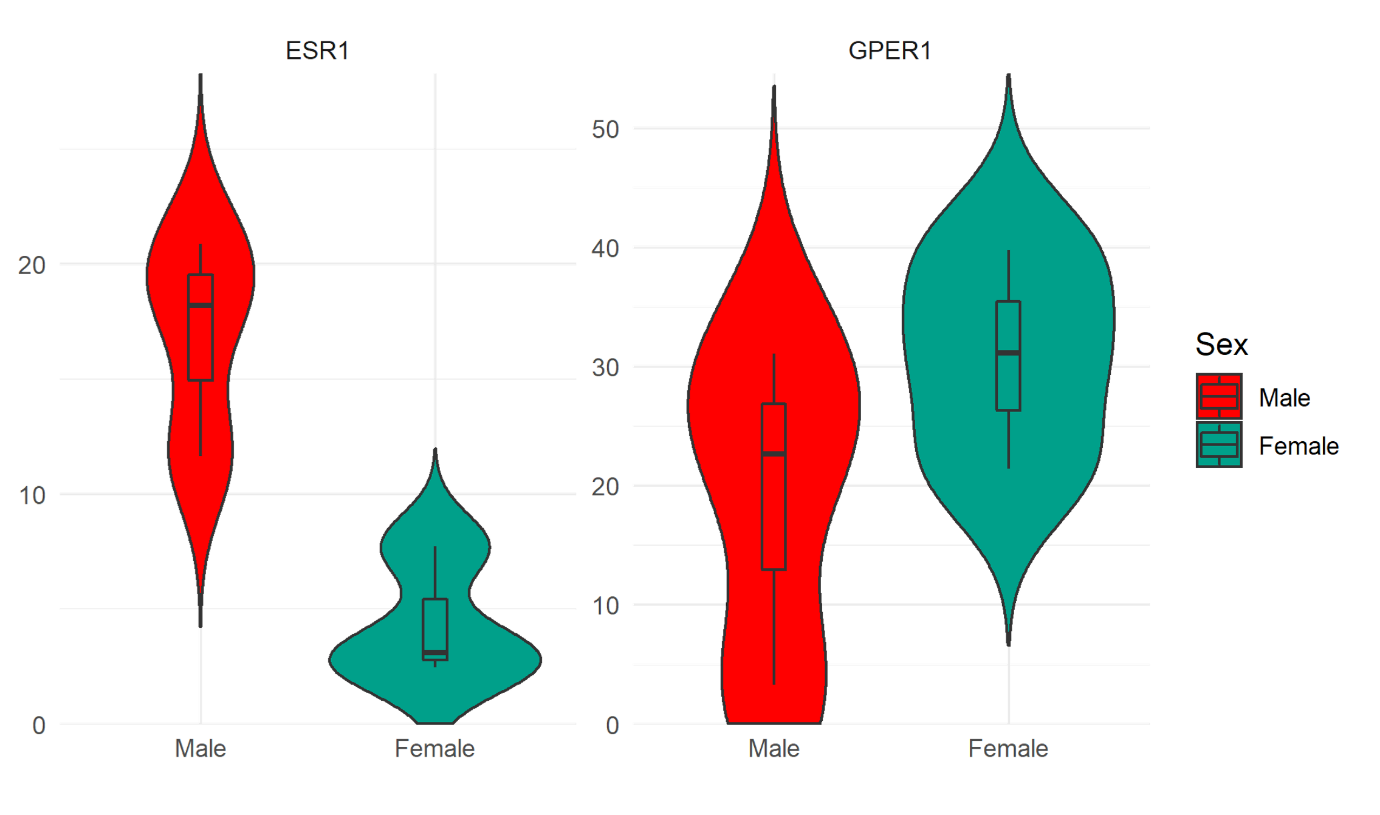


Fig. S3. Oestrogen receptor expression in MGLs: Violin and box plots showing the kernel probability, the median, and interquartile range of the normalized expression counts of ESR1 and GPER1.


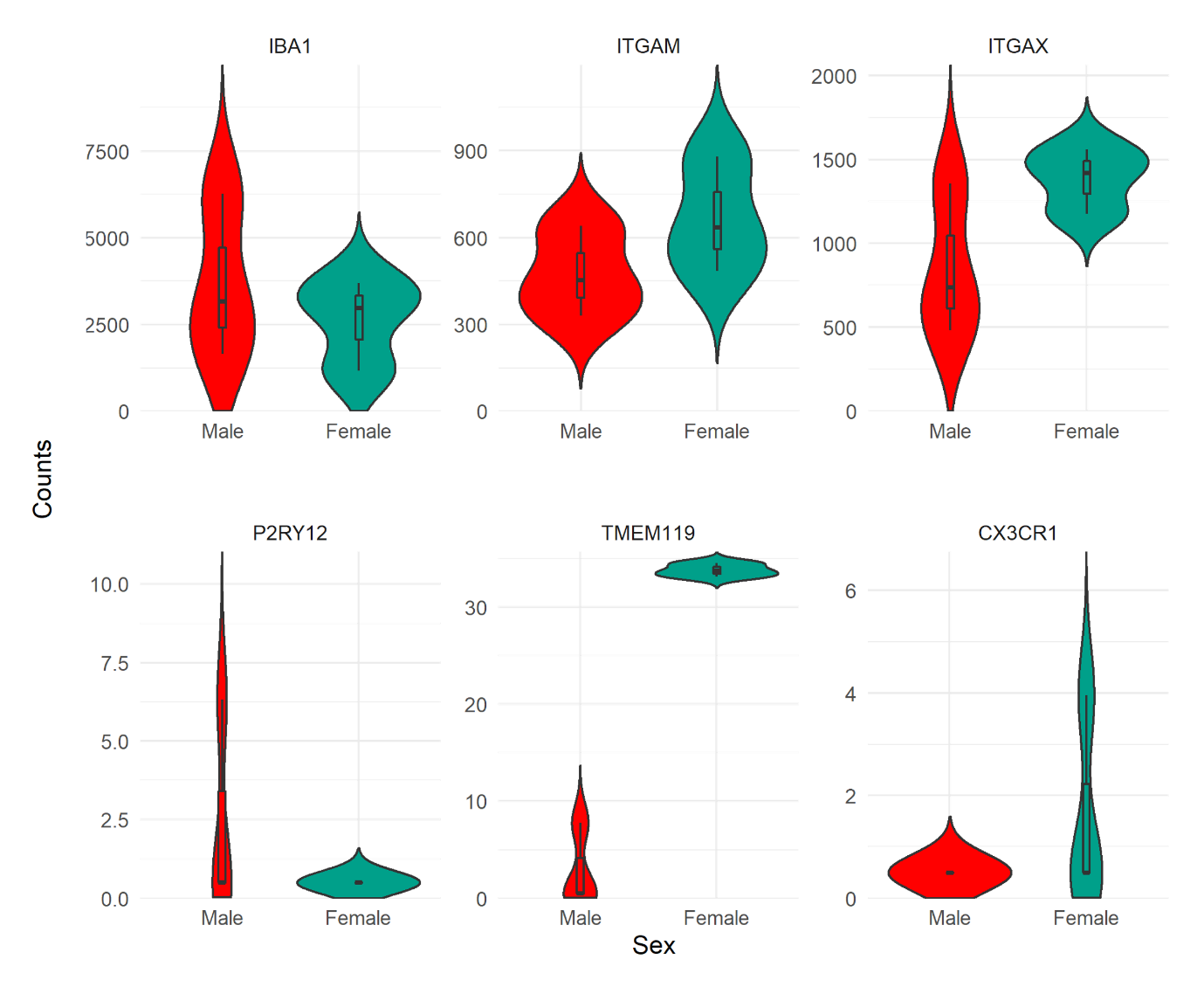


Fig. S4. Expression of microglial genes in MGLs: Violin and box plots of the normalized expression counts of key microglial genes, including IBA1/AIF1, ITGAM (CD11b), ITGAX (CD11c), P2RY12, TMEM119, and CX3CR1 in male and female induced pluripotent stem cell-derived microglial-like cells (MGLs).

**Table S1:** Sexually differentially expressed genes in the microglial nuclei.

**Table S2:** Genes comprising each of the IR-AD gene sets

**Table S3:** Sexually differentially expressed genes between the F- and M-MCs

**Table S4:** GSVA of IR-AD gene sets between F_lo_- and F_hi_-MDMs results

**Table S5:** Sexually differentially expressed genes between the F_lo_- and M-MDMs.

**Table S6:** Sexually differentially expressed reactome pathways between the F_lo_- and M-MDMs.

**Table S7:** Differentially expressed reactome pathways between the F_lo_- and F_hi_-MDMs.

**Table S8:** Smith et al., (2021) Sample specific metadata.

**Table S9:** Reactome gene sets used for the analysis of the MDMs.
